# Genomic evidence for sulfur intermediates as new biogeochemical hubs in a model aquatic microbial ecosystem

**DOI:** 10.1186/s40168-021-00999-x

**Published:** 2021-02-16

**Authors:** Adrien Vigneron, Perrine Cruaud, Alexander I. Culley, Raoul-Marie Couture, Connie Lovejoy, Warwick F. Vincent

**Affiliations:** 1 Département de Biologie, Université Laval, Québec, QC Canada; 2 Centre d’études nordiques (CEN), Takuvik Joint International Laboratory, Université Laval, Québec, QC Canada; 3 Institut de Biologie Intégrative et des Systèmes, Université Laval, Québec, QC Canada; 4 Département de Biochimie, de Microbiologie et de Bio-informatique, Université Laval, Québec, QC Canada; 5 Département de Chimie, Université Laval, Québec, QC Canada; 6 Québec Océan, Université Laval, Québec, QC Canada

**Keywords:** Meromictic lakes, Anoxic basin, Arctic Ocean, Sulfur cycling, Organic sulfur, Sulfur intermediates, Redox gradients, Metagenomics

## Abstract

**Background:**

The sulfur cycle encompasses a series of complex aerobic and anaerobic transformations of S-containing molecules and plays a fundamental role in cellular and ecosystem-level processes, influencing biological carbon transfers and other biogeochemical cycles. Despite their importance, the microbial communities and metabolic pathways involved in these transformations remain poorly understood, especially for inorganic sulfur compounds of intermediate oxidation states (thiosulfate, tetrathionate, sulfite, polysulfides). Isolated and highly stratified, the extreme geochemical and environmental features of meromictic ice-capped Lake A, in the Canadian High Arctic, provided an ideal model ecosystem to resolve the distribution and metabolism of aquatic sulfur cycling microorganisms along redox and salinity gradients.

**Results:**

Applying complementary molecular approaches, we identified sharply contrasting microbial communities and metabolic potentials among the markedly distinct water layers of Lake A, with similarities to diverse fresh, brackish and saline water microbiomes. Sulfur cycling genes were abundant at all depths and covaried with bacterial abundance. Genes for oxidative processes occurred in samples from the oxic freshwater layers, reductive reactions in the anoxic and sulfidic bottom waters and genes for both transformations at the chemocline. Up to 154 different genomic bins with potential for sulfur transformation were recovered, revealing a panoply of taxonomically diverse microorganisms with complex metabolic pathways for biogeochemical sulfur reactions. Genes for the utilization of sulfur cycle intermediates were widespread throughout the water column, co-occurring with sulfate reduction or sulfide oxidation pathways. The genomic bin composition suggested that in addition to chemical oxidation, these intermediate sulfur compounds were likely produced by the predominant sulfur chemo- and photo-oxidisers at the chemocline and by diverse microbial degraders of organic sulfur molecules.

**Conclusions:**

The Lake A microbial ecosystem provided an ideal opportunity to identify new features of the biogeochemical sulfur cycle. Our detailed metagenomic analyses across the broad physico-chemical gradients of this permanently stratified lake extend the known diversity of microorganisms involved in sulfur transformations over a wide range of environmental conditions. The results indicate that sulfur cycle intermediates and organic sulfur molecules are major sources of electron donors and acceptors for aquatic and sedimentary microbial communities in association with the classical sulfur cycle.

**Video abstract**

**Supplementary Information:**

The online version contains supplementary material available at 10.1186/s40168-021-00999-x.

## Background

The sulfur cycle encompasses complex energetic processes where sulfur (S) ions and molecules in different oxidation states, from the most reduced (− 2: sulfides, H_2_S/HS^−^) to the most oxidised (+ 6: sulfate, SO_4_^2−^), are transformed through oxidation, reduction, disproportionation [1] and putative comproportionation [2] by taxonomically diverse microorganisms. The sulfur cycle is tightly interwoven with carbon, nitrogen and metal cycles, and is tied to both cellular and ecosystem-level processes [3]. In marine sediments, sulfate is an ubiquitous electron acceptor and sulfate-reducing microorganisms have been estimated to contribute to up to 29% of organic matter remineralisation in aquatic environments [4]. Sulfate reducers can generate massive concentrations of sulfides, which in turn serve as an electron donor for symbiotic or free-living sulfur-oxidizing microorganisms that recycle sulfides into sulfate [5].

Although sulfate reduction and sulfide oxidation have received the most attention, our knowledge of the identity of microorganisms and metabolic pathways involved in these processes is limited and novel lineages of microorganisms mediating steps in the sulfur cycle remain to be discovered [6, 7]. Furthermore, sulfur cycling is highly complex; sulfur transformations by microorganisms and the geochemical reactivity of reduced sulfur molecules with metal oxides generate several inorganic sulfur compounds of intermediate oxidation states such as thiosulfate (S_2_O_3_^2−^), tetrathionate (S_4_O_6_^2−^), sulfite (SO_3_^2−^), and polysulfides (S^2−^_n+1_). These inorganic S compounds are all substrates for further microbial oxidation, reduction or disproportionation [3, 8] and precipitation with reduced metals [9]. The rapid recycling of these sulfur species has biogeochemical significance, especially in low sulfate environments, where the rapid turn-over of these compounds provides an opportunity for shortcuts in the sulfur cycling and potentially sustains a large variety of microorganisms [10]. However, because efficient microbial use drives their concentrations down to the picomolar range, the importance of these compounds and the associated microbial processes remain unrecognised. An alternative strategy to uncover such processes is to look for genomic evidence of the identity, ecology and functional properties of microbes metabolizing these compounds.

The genetic underpinning of sulfur transformations is still poorly resolved and is complicated by the bidirectional activities of key enzymes and by the diversity and complexity of many enzymatic pathways. For example, the metabolic pathways involved in sulfur compounds disproportionation remains unknown and even the exact mechanisms involved in sulfide oxidation remain little understood [1, 11]. Another important, yet largely overlooked component of the sulfur cycle involves the utilisation and formation of organo-sulfur molecules (OSM). These labile metabolites, including sulfonate (compounds with a R-SO_3_^−^ functional group) and sulfonium such as dimethylsulphoniopropionate (DMSP), are produced by macro- and micro-algae and may represent an important source of sulfur for certain microorganisms in freshwaters [12, 13] and oceans, where the genetic potential for transformation of OSM is widespread in marine bacteria [14].

Due to the abundance of sulfur compounds in sea water, much of our knowledge about sulfur cycles comes from marine sediments, which constitute a major biotope for sulfur cycling microorganisms [3]. However, the distribution of sulfur cycling microorganisms is not limited to marine environments, and sulfur cycling microorganisms also have important ecological roles in environments with low sulfate concentrations such as wetlands and lakes [7, 15].

Marine-derived lakes are natural laboratories for understanding the sulfur cycle. In the polar regions, melting ice sheets lead to an isostatic rebound of the continents, isolating fjords that become seawater-trapped lakes. Melting snow and glaciers then discharge freshwater into these lakes that float on the denser seawater beneath. This density gradient is re-enforced by a mostly permanent ice cover, resulting in meromictic lakes that are layered physically and chemically with well-defined environments, enabling the development of complex and stable microbial communities along light, nutrient and redox gradients [16]. The water column of these lakes is divided into a mixolimnion consisting of the oxic surface freshwater layer immediately beneath the ice, and an anoxic and sulfidic monimolimnion, derived from the marine bottom layer. The chemocline at the interface between these layers is a zone of highest chemical reactivity in the lake, typically associated with elevated microbial activity and intense sulfur cycling [17, 18]. Phototrophic or chemotrophic sulfur oxidisers that recycle sulfides produced in the anoxic monimolimnion overlap or coexist in the chemocline, depending on oxygen and light penetration [19]. The activity of these microorganisms results in the production of sulfate and sulfur deposits outside the cells, leading to yellow or orange coloration of the water [20]. In addition, metal oxides (iron and manganese) frequently accumulate in meromictic lakes [21], leading to chemical oxidation of sulfur species.

The broad range of physico-chemical conditions in a single water column makes polar meromictic ecosystems ideal lake-size laboratories to investigate sulfur-cycling microorganisms and metabolic pathways. We hypothesised that sulfur cycle intermediate (SCI) molecules such as thiosulfate, tetrathionate, sulfite and polysulfides are a major source of energy for aquatic microbial communities, and that related biogeochemical processes would be evident in a polar meromictic lake because of the prolonged selection of microbial species and functions across stable biogeochemical gradients.

**Fig. 1:**
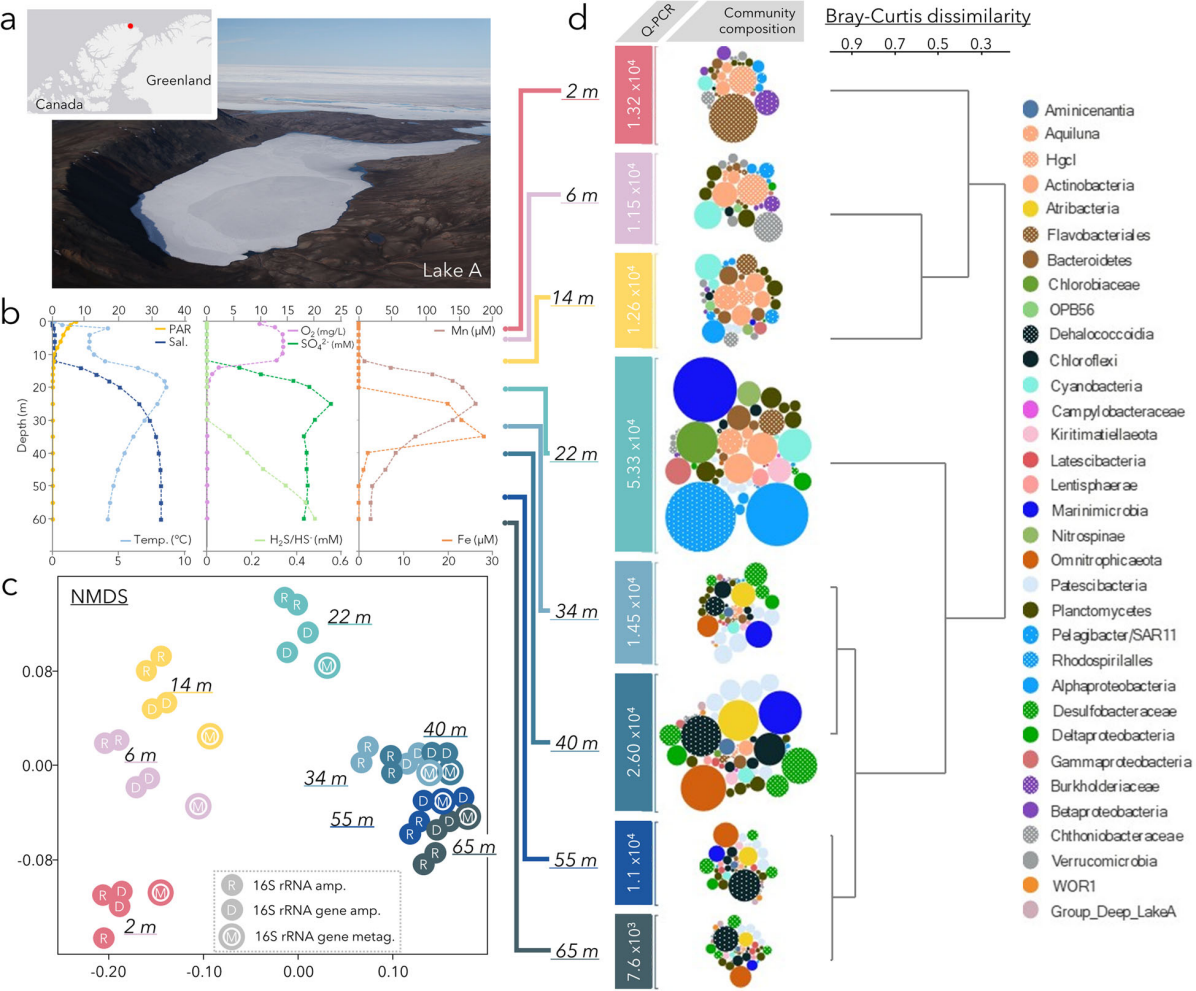
Overview of the physicochemical and microbial features of Lake A. **a** General location and photograph of Lake A. **b** Physicochemical profiles in the lake. Photosynthetically active radiation data are derived from Charvet et al. 2012, SO_4_^2−^, H_2_S, total Mn and total Fe were extrapolated from Gibson et al. 2002. **c** NMDS of the bacterial community based on Bray-Curtis dissimilarity index calculated with 16S rRNA amplicons and metagenomic datasets. **d** Relative bacterial abundance (qPCR) and community composition based on 16S rRNA genes from metagenomes. The size of the circles represents the relative abundance of genes for each lineage. Only lineages with relative proportions > 1% of the reads in at least one sample are shown. Overall size of the community and associated qPCR bar is related to qPCR data. qPCR data are expressed in number of 16S rRNA genes per mL of lake water

To test this hypothesis, we analysed the microbial community composition, abundance and metabolic potential in Lake A (83°00′N, 75°30′W), a deep marine-derived meromictic lake in the Canadian High Arctic (Fig. 1a), with a focus on sulfur cycling microorganisms across light and redox gradients. Applying genome-centric metagenomics, we recovered a large variety of sulfur cycling microorganisms with contrasting and complex metabolic pathways involved in sulfur transformations. We elucidated the importance of sulfur cycle intermediates as well as organic sulfur molecules as potential energy sources for microorganisms over the broad range of geochemical conditions that are represented in this model biogeochemical system.

## Results

Oxygen, salinity and temperature profiles of Lake A, measured at the sampling time (18 July 2017), showed that the water column under the ice was highly stratified, with similar profiles to previous years [21]. The freshwater oxygenated mixolimnion extended to the chemocline, located from 12 to 24 m below the upper ice surface ice, and marine-derived saline, anoxic water occurred below 24 m (Fig. 1b).

### Microbial community composition

Microbial community composition and abundance was investigated by 16S rRNA and 16S rRNA gene sequencing and quantification (qPCR) down the water column with discrete samples from 2, 6, 14, 22, 34, 40, 55, and 65 m. In addition, shotgun metagenomes were also sequenced from each water layer and 16S rRNA gene sequences were extracted from the metagenomic dataset and analysed along with the amplicon data to detect any microbial lineages that might have been missed due to PCR primer bias (Fig. 1d). Bacteria represented nearly 100% of the 16S rRNA genes in both qPCR and metagenomic data in the upper freshwater samples and 90% in the saline water samples (Supplementary Figure S1); therefore, only the bacterial community is detailed in this study. The bacterial community compositions of replicate samples were highly similar (Fig. 1c and Supplementary Figure S1). Although the number of rRNA genes may fluctuate depending on species and physiological state, detection of 16S rRNA was used as a proxy for identifying metabolically active microorganisms [22]. Taxonomic profiles from 16S rRNA and 16S rRNA genes were largely congruent, with 92% of the bacterial lineages being detected using these two approaches, suggesting that these communities were likely active (Supplementary Figure S2). The results were also congruent with the microbial community composition recovered from metagenomic data with the exceptions of SAR11 (up to 3.8% and 1.5% of the 16S rRNA genes in metagenomes and amplicons respectively) and *Patescibacteria* (14.8% and 4.4% of the 16S rRNA genes in metagenomes and amplicons respectively) lineages, which appeared to be underestimated by amplicon sequencing (Fig. 1c and Supplementary Figure S2).

The different depth strata of the lake had sharply contrasting community structures (non-parametric multivariate analysis of variances, NPMANOVA, *p =* 0.0001, Fig. 1c, d). The bacterial community from the 2 m sample collected immediately beneath the ice was dominated by *Flavobacteriales* (39% of the 16S rRNA metagenomic reads), *Burkholderiales* (10%) and *Actinobacteria* HgcI (13%). At 6 m, *Cyanobacteria* (12%), *Verrucomicrobia* (*Chtoniobacteraceae*, 14%) and *Actinobacteria* HgcI (16%) were predominant. At the freshwater-saline transition (14 m), the proportion of *Alphaproteobacteria* (7%), including SAR11, *Planctomycetes* (4%) and *Bacteroidetes* (10%) increased. At the bottom of the oxic-anoxic transition zone (22 m), the microbial community was dominated by *Alphaproteobacteria* (13%), including members of the *Rhodospirillalles* (17%), *Marinimicrobia*, also known as SAR406 (14%), *Chlorobiaceae* (6%) and *Cyanobacteria* (4%). In the anoxic saline strata of the lower water column (34, 40, 55 and 65 m), the microbial community was mainly dominated by *Chloroflexi* (up to 19% at 55 m), *Desulfobacteraceae*, including the SEEP SRB1 lineage (10%), *Atribacteria* (up to 9% at 40 m), *Patescibacteria* (up to 15% at 34 m) and *Omnitrophicaeota* (up to 12% at 65 m). The proportion of unclassified reads also increased with depth, from only 4% of the reads in surface water to 40% at 65 m in the 16S rRNA gene amplicon datasets (Supplementary Figure S1). However, up to 28% of the unclassified reads of the 65 m samples were related to a single operational taxonomic unit (OTU, 97% similarity) distantly related (96% sequence similarity) to a sequence recovered from the Okinawa Trough. Quantification of 16S rRNA genes indicated a relatively constant bacterial abundance throughout the water column, with on average 1.36 ± 0.6 × 10^4^ 16S rRNA genes ml^−1^, except for the oxic-anoxic transition zone where bacterial abundance reached 5.33 × 10^4^ 16S rRNA genes ml^−1^ (Fig. 1d).

### Depth distribution of sulfur cycling genes

**Fig. 2:**
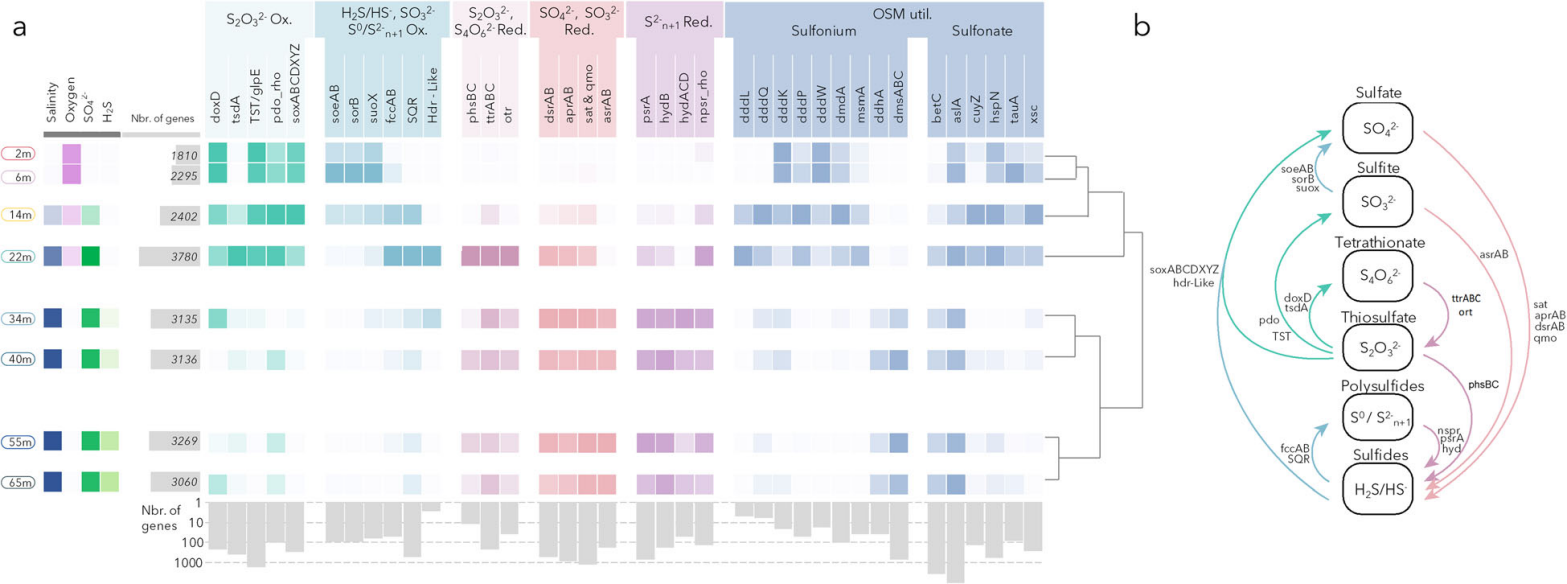
Distribution of sulfur cycling genes. **a** Vertical profiles of sulfur cycling genes identified in metagenomes along geochemical gradients (heat map of salinity, oxygen, SO_4_^2−^ and H_2_S). Hierarchical clustering at the right was computed based on all KEGG orthologues identified in metagenomes. Numbers of genes per sample and per gene are after normalization. **b** Schematic representation of the sulfur cycle. Names of the enzymes are available in Supplementary Table S1

Shotgun metagenomes from the eight depths were sequenced to evaluate the metabolic potential of Lake A microbial communities. A total of 10,378 different genes (KEGG Orthologues) were identified in the metagenomes. Hierarchical clustering of the overall metabolic potentials (all detected KOs) of the samples was congruent with the clustering based on taxonomic profiles and geochemical data, with two major clusters: the freshwater samples (2, 6 and 14 m) and the saline anoxic samples (34, 40, 55 and 65 m); and the oxycline sample (22 m) branching separately on the dendrogram (Fig. 2). A large number of genes involved in sulfur cycling were identified in all samples and were distinctly distributed vertically according to the physicochemical gradients (Fig. 2).

Genes for thiosulfate oxidation (*soxABCDXYZ*, *doxD*, *tsdA*, *TST* and *pdo*_Rho, corresponding to a gene of persulfide dioxygenase fused with a rhodanese domain) were detected in all freshwater samples (from 2 to 22 m). Hydrogen sulfide (*soxABCDXYZ*) and sulfite (*soeAB*, *suoX*) oxidation genes were mainly identified in the most oxygenated freshwater layers, whereas genes for sulfide (*fccAB*), and polysulfide (*SQR* and *Hdr*-like, a novel gene involved in bacterial sulfur oxidation [23]) oxidation pathways were mainly found in the micro-oxic conditions of the oxycline waters (14 and 22 m) (Fig. 2). Few of these genes were also detected in anoxic waters. By contrast, genes involved in dissimilatory sulfate and sulfite reduction pathways (*dsrAB*, *aprAB*, *sat*, *qmo*, *asrAB*) were mainly detected in saline and anoxic waters (34, 40, 55 and 65 m). Polysulfide and thiosulfate reductases (Psr and Phs) are complex iron–sulfur molybdoenzymes with such a close phylogenetic relationship and with so few characterised representatives, that distinction based on sequence of the catalytic unit (phsA-psrA) is currently impossible [24]. Here, we used the more characterised B and C subunits of the thiosulfate reductase (phsBC), encoding the electron transporter and anchor as a proxy of the entire thiosulfate reducing Phs complex. Although anchor proteins could be involved in other functional pathways, phsBC and ttrBC, are among the rare genes of anchor and electron transfer protein characterised in Kegg orthology suggesting evidence that these KOs are associated with their described functions [25]. Furthermore, in absence of phsBC genes, we considered the presence of psrA-phsA gene as an indicator of the polysulfide reductase complex psrABC rather than phsABC. Given the limited information about these genes, we considered this to be a pragmatic and best supportable approach. As sulfate and sulfite reduction pathways, thiosulfate (*phsBC*) and tetrathionate (*ttrABC*, *otr*) reductions and polysulfide and elemental sulfur reductions (*psrA*, *hydABCD*, *npsr*) were detected in saline and anoxic waters. All oxidative and reductive pathways were detected at the bottom of the oxycline (22 m) (Fig. 2).

Genes for the transformation of organic sulfur molecules (sulfonium and sulfonate) were also identified (Fig. 2). Genes involved in degradation of dimethylsulfoniopropionate (*dddLQKPW*, *dmdA*), methanesulfonate (*msmA*) and other sulfonates, such as cysteine (*cuyAZ*), sulfopropanediol (*hspN*), taurine (*tauA*) or sulfoacetaldehyde (*xsc*) were detected throughout the water column but in higher proportion in freshwater. By contrast, genes coding dimethyl sulfoxide reductases (*dmsABC*) were mainly found in anoxic saline waters (Fig. 2). Genes for sulfatases (*betC* and *arslA*), catalysing hydrolysis of sulfate esters, were abundant throughout the water column, with higher numbers at the bottom of the oxycline (22 m) (Fig. 2).

### Metagenome assembled genomes of S-cycling populations

**Fig. 3:**
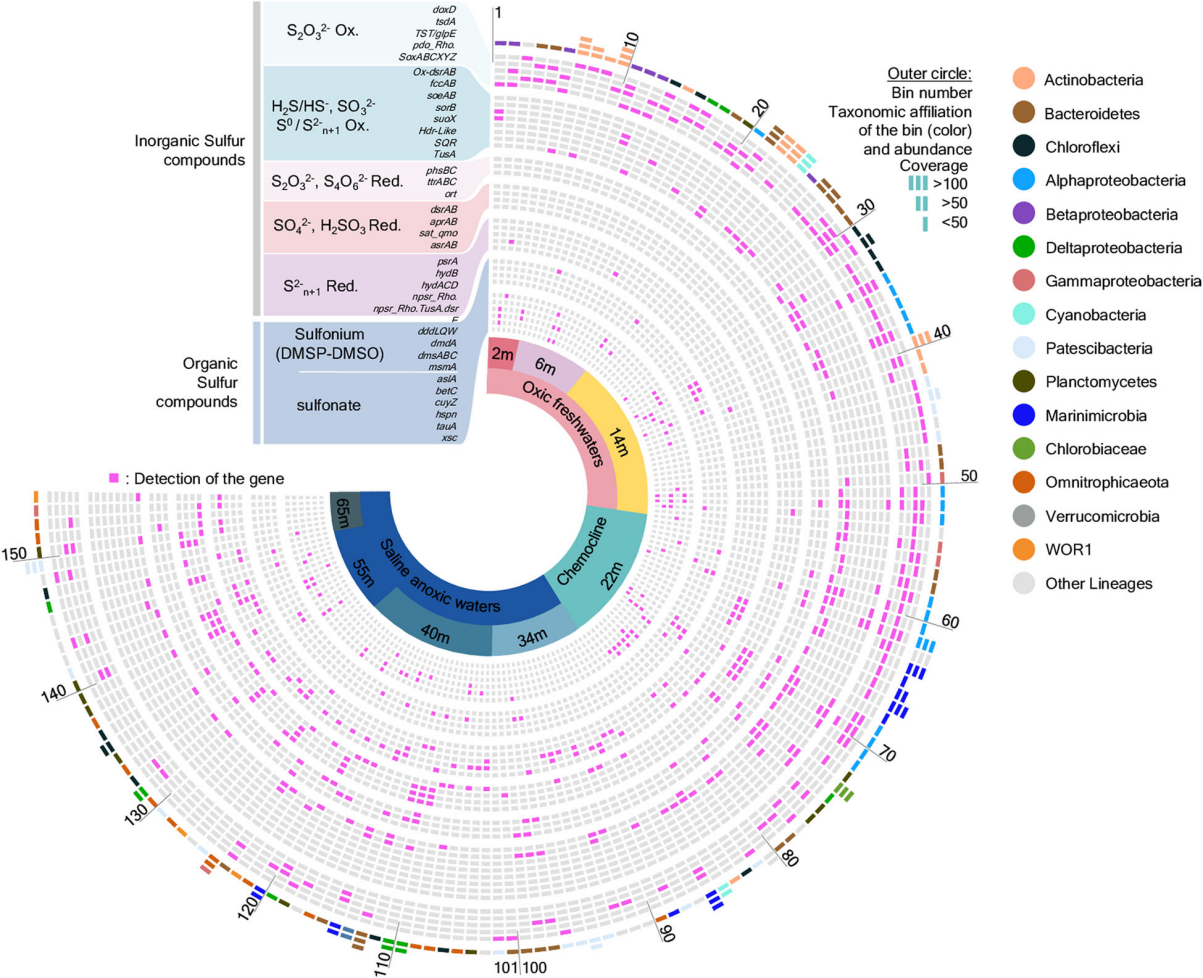
Key genes for inorganic and organic sulfur transformation identified in the 154 genomic bins with sulfur cycling genes. Colors in the outer circle represent the taxonomic affiliations of the bins. Bins are sorted per depth where they have been recovered. Ox. Oxidation Red. Reduction. Details are provided in supplementary Table S1

A total of 250 genomic bins with > 50% completeness and < 5% contamination levels were recovered from the combined metagenomic dataset (Supplementary Table 1, Figure S4). Among them, 154 genomic bins (61.4%) harboured genes for sulfur cycling (Fig. 3). Taxonomic affiliation of these bins, inferred from 16S rRNA gene and other ribosomal protein sequences, indicated a large diversity of sulfur cycling microorganisms representing most of the predominant lineages identified in the water column by 16S rRNA gene approaches. The most represented lineages in bins with sulfur transformation genes (Fig. 3) were *Bacteroidetes* (21 different bins), *Alphaproteobacteria* (17 bins), *Parcubacteria* (17 bins), *Omnitrophicaeota* (12 bins), *Chloroflexi* (12 bins), *Actinobacteria* (11 bins), *Deltaproteobacteria* (9 bins), *Planctomycetes* (9 bins) and *Marinimicrobia* (8 bins).

#### Sulfur-oxidizing populations

**Fig. 4:**
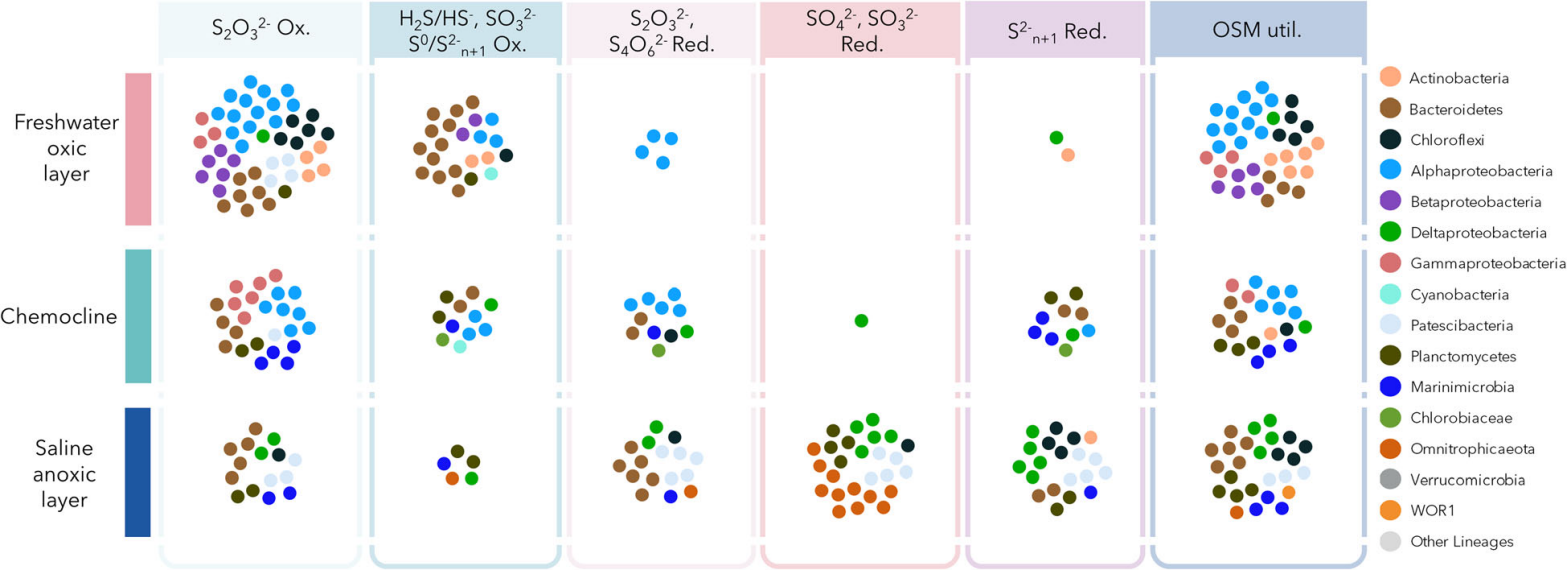
Depth distribution of metabolic potentials identified in the genomic bins. Each point represents a genomic bin with the color corresponding to its taxonomy

The potential for thiosulfate oxidation was widespread in the community (Figs. 3 and 4). Numerous *Parcubacteria*, *Actinobacteria* and *Chloroflexi* bins included genes for the thiosulfate:quinone dehydrogenase (*doxD*), while the gene for the thiosulfate dehydrogenase (*tsdA*) was mainly detected in *Alphaproteobacteria* and *Bacteroidetes.* Genes of persulfide dioxygenase fused with a rhodanese domain (*pdo_rho*), potentially involved in thiosulfate oxidation [26] were widespread in the *Bacteroidetes* and *Planctomycetes* bins (Fig. 3). The SoxABCDXYZ complex for thiosulfate oxidation was also identified in *Alpha*- and *Beta*-*proteobacteria* (Fig. 3). In addition to Sox genes, oxidative DsrAB genes were identified in *Alphaproteobacteria*, indicating potential for H_2_S oxidation to sulfate. Oxidative DsrAB genes and genes for sulfide (*fccAB*) and sulfur oxidation through the sulfide:quinone oxidoreductase (SQR) were identified in the *Chlorobiaceae* as well as in few *Alphaproteobacteria* bins. *Betaproteobacteria* bins harboured genes for sulfite oxidation (*soeAB* and *sorB*). The SQR gene was also detected in *Cyanobacteria*, *Actinobacteria*, *Bacteroidetes*, *Gamma*- and *Deltaproteobacteria*. Sulfur and thiosulfate oxidation through a Hdr-like complex [23] was identified in one alphaproteobacterial bin.

#### Sulfur-reducing populations

The potential for thiosulfate reduction through thiosulfate reductase (PhsBC genes) was identified in *Bacteroidetes* bins recovered from anoxic samples (Figs. 3 and 4). Tetrathionate reductase genes (*ttrABC*) were mainly found in *Rhodospirillales* bins while genes coding the octoheme tetrathionate reductase (*otr*), involved in tetrathionate and potentially nitrite and hydroxylamine reduction [27] (Supplementary Figure S3), were detected in few *Desulfobacteraceae*, *Anaerolineae*, *Chlorobiaceae* and *Bacteroidetes* bins. The sulfate reduction pathway (DsrAB, AprAB, Sat and Qmo genes) was identified in *Deltaproteobacteria* bins as well as in one *Chloroflexi*, two *Planctomycetes* and in *Candidatus* Nealsonbacteria, *Ca*. Zixibacteria and *Ca*. Abyssubacteria bins recovered from the saline anoxic waters (Figs. 3 and 4). Anaerobic sulfite reductase genes (*asrAB*) were detected in all *Omnitrophica* bins as well as in two *Planctomycetes* and two *Parcubacteria* bins. Polysulfide reductase genes (*psrA*) were found in *Deltaproteobacteria* as well as in some *Marinimicrobia* and *Bacteroidetes* bins. Sulfhydrogenase genes (*hydABCD*), involved in S^0^ and polysulfide reduction were also identified in genomic bins. However, while HydACD genes were detected in 8 *Parcubacteria* bins, including *Ca*. Kuenenbacteria, the gene coding the beta subunit (*hydB*) was detected only in half of these bins. By contrast *hydB* alone was also detected in *Omnitrophica*, *Marinimicrobia* and WOR1 (*Ca.* Saganbacteria) bins (Fig. 3). NADH-dependent persulfide reductase genes (*npsr*), involved in persulfide, polysulfide or S^0^ reduction were identified in *Deltaproteobacteria*, *Ca*. Abyssubacteria and *Calditrichaeota* as well as in two *Planctomycetes* bins. A fusion of DsrE, Npsr and TusA genes and including two rhodanese regions was also identified in the metagenomic sequences and in two bins related to *Ca*. Nealsonbacteria and *Phycisphaeraceae* (Fig. 3), suggesting a novel gene. Rhodanese regions and TusA protein are involved in sulfur binding and intracellular transport while both DsrE and Npsr are involved in sulfur reduction, suggesting a role in intracellular elemental sulfur reduction for this new gene.

#### Organic sulfur utilizing populations

**Fig. 5:**
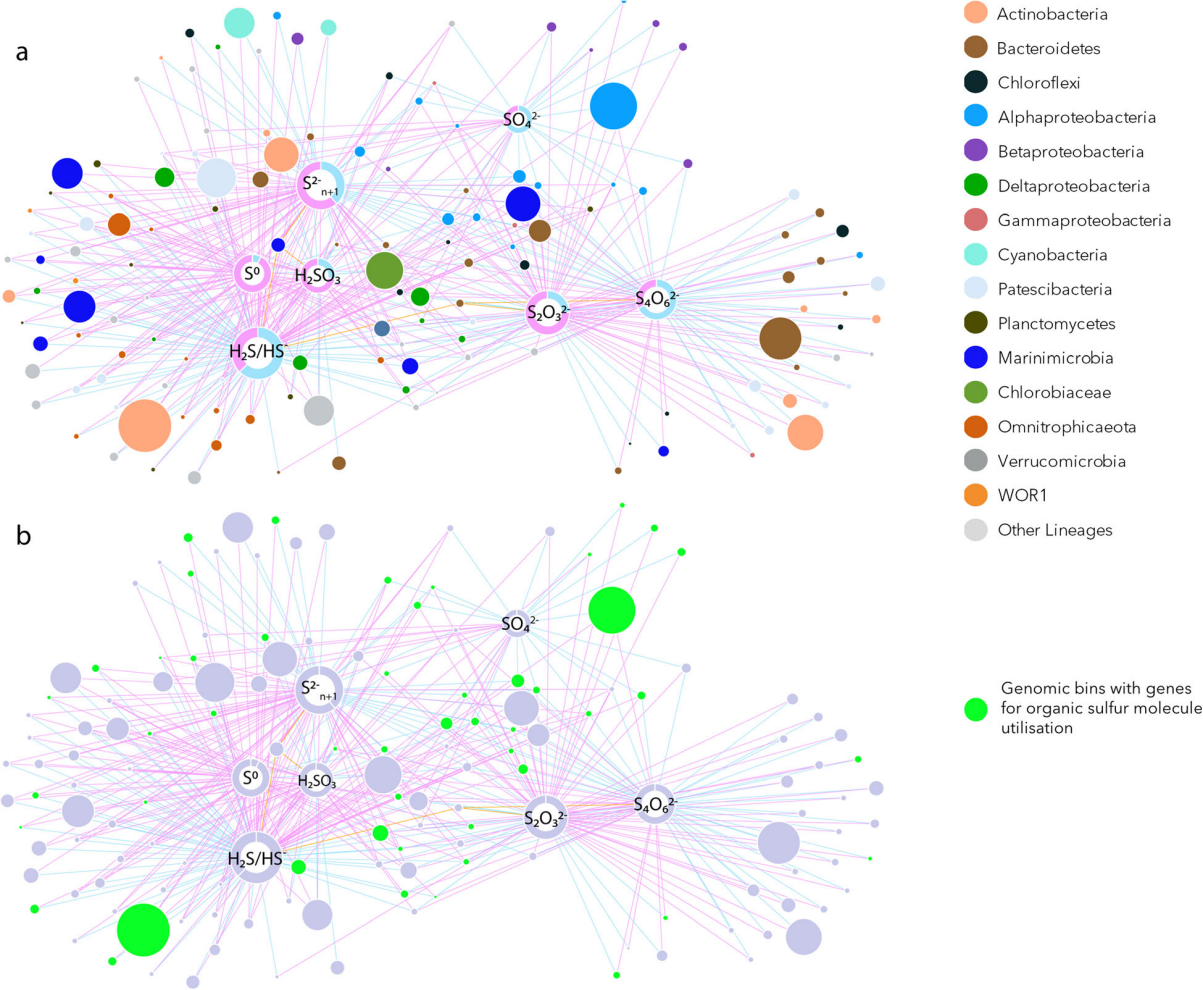
Sulfur cycling network of the Lake A microbiome. **a** Genomic bins are represented as circles with color corresponding to their taxonomic affiliation and size corresponding to the average number of reads mapping on the genomic bin. Genomic bins are connected to sulfur compounds (white circle with pie chart) predicted to be utilised (blue link) or produced (purple link). For each sulfur molecule, the ring pie chart represents the proportion of consumption (blue) and production (purple) and the size of the pie chart is proportional to the number of connections. **b** Metabolic network overlaid with the potential for organic sulfur molecule utilisation in green

A metabolic potential for organic sulfur utilisation was detected in 87 bins, mainly related to proteobacterial lineages, was recovered throughout the water column (Fig. 4). The potential for transformation of dimethylsulfoniopropionate (DMSP) into dimethylsulfide (DMS) was detected in *Alphaproteobacteria* bins, including SAR11/*Pelagibacter* bin (Figs. 3 and 4). By contrast, genes for anaerobic dimethylsulfoxide reductase (*dmsAB*) were identified in *Deltaproteobacteria*, *Chloroflexi* and few *Bacteroidetes* bins. The metabolic potential for sulfonate degradation, including genes coding for enzymes involved cysteine (*cuyAZ),* sulfopropanediol (*hspN*), taurine (*tauA*) and sulfoacetaldehyde (*xsc*) catabolism, was mainly found in *Actinobacteria*, *Planctomycetes* and *Alpha*- and *Beta*-*proteobacteria* bins (Figs. 3, 4 and 5). Multiple (more than 10) sulfatase genes were identified in some *Planctomycetes*, *Bacteroidetes*, *Alphaproteobacteria* and *Chloroflexi* bins.

### Microbial network for sulfur metabolism

Based on the identified genes, metabolic capabilities of Lake A populations were inferred to determine which S-containing molecules were potentially produced or consumed throughout the water column (Fig. 5). Up to 100 different bins (65% of the bins with S-cycling genes) were predicted to be associated with hydrogen sulfide production or consumption. Metabolic potentials involving the production or consumption of polysulfides (89 bins, 58%), thiosulfate (72 bins, 47%) and tetrathionate (61 bins, 40%) were also widespread in the Lake A community (Fig. 5). A metabolic potential associated with sulfite and sulfate was less represented in the community with only 46 bins (30%) associated with sulfite and 30 bins (19%) with sulfate (Fig. 5).

## Discussion

Ice-capped, permanently stratified Lake A is an extreme microbial ecosystem, where strong, persistent environmental gradients provide a natural model for broader understanding of aquatic biogeochemical cycles. The water physico-chemical stratification profile measured in 2017 in Lake A has been observed since 1974 [21, 28], indicating a highly stable system and allowing extrapolation of geochemical profiles from historical data. Based on a previous complete geochemical characterization of the lake waters [21], high concentrations of sulfate occur in the chemocline and in the anoxic waters, where sulfide concentrations increase with depth (Fig. 1). High manganese concentrations (total Mn) were also detected at the chemocline, with a peak of iron (total Fe) a few meters below (Fig. 1 [21]). Finally, under similar conditions of snow and ice cover, photosynthetically available radiation (PAR) was detected down to 20 m (Fig. 1 [28]). These extrapolations were supported by the depth distribution of green sulfur bacteria (*Chlorobium*) that confirmed light and sulfide transition zones around 24 m. Together, these observations indicate a stable and intense redox gradient throughout the water column for microbial selection and growth.

DNA- and RNA-based amplicon sequencing was initially carried out to determine the composition of the total and potentially active microbial community in the water column. The results of the amplicon sequencing revealed that each depth sampled had a unique microbial community composition, and we therefore sequenced metagenomes for all depths. We extracted 16S rRNA genes from metagenomes to detect any microbial lineages that might have been underestimated due to PCR primer bias. Since we found few discrepancies, we have chosen to present here the microbial community detected by the metagenomic analyses. The amplicon results remain valuable as indicators of potential activity and facilitated comparisons of our data with more commonly reported amplicon data from elsewhere. Finally, we extended our analyses with quantitative PCR since neither the amplicon nor metagenomic approaches provided estimations of abundances.

The ensemble of RNA and DNA-based 16S amplicon and metagenomic sequencing from High Arctic Lake A revealed multiple contiguous layers of complex yet stable and potentially active microbial communities, with putative metabolism aligning with the geochemical gradients of the lake (Fig. 1). The microbial community of the oxic mixolimnion beneath the ice was consistent with cold freshwater communities, with lineages of the *Verrucomicrobia*, *Bacteroidetes*, *Actinobacteria*, *Cyanobacteria* and *Betaproteobacteria*, which are frequently observed in northern lakes and rivers [29–31]. At the chemocline, alphaproteobacterial chemotrophic sulfur oxidisers and phototrophic sulfur oxidisers (*Chlorobiaceae*), both previously observed in microbial surveys of Antarctica [17, 32] and temperate meromictic lakes [19, 33] co-occurred since the lower depth limits of the Lake A photic and aerobic zones coincided (Fig. 1). The chemocline microbial community also shared major similarities with marine communities with, for example, high proportions of *Marinimicrobia* (SAR406), *Pelagibacter* (SAR11) and *Deltaproteobacteria* SAR324, which are frequently detected in seawater, hadal waters in the deep ocean, dysoxic marine waters and oxygen minimum zones [24, 34–36]. By contrast, the microbial community of the saline anoxic monimolimnion showed homologies with deep-sea hypersaline anoxic basin communities, notably with sequences related to *Chloroflexi* MSBL5, *Desulfobacteraceae* MSBL7, *Planctomycetes* MSBL9 and *Cloacimonadales* MSBL8 [37]. At these depths, the microbial community also shared similarities with anoxic and sulfidic marine sediments, where *Deltaproteobacteria* SEEP SRB1 and *Desulfarculaceae*, *Atribacteria*, *Omnitrophica* and *Chloroflexi* members also flourish [38]. Taken together, these results reveal that, cascading along its geochemical gradients, the Lake A water column hosts a panoply of microorganisms, relevant to a broad range of environments and environmental conditions from oxic freshwaters to anoxic marine sediments. It is rare for a single study to cover the entire range of freshwater oxic to marine anoxic conditions. In this way, we were able to track amplicons and MAGs that had peaks in one environment but were able to persist in adjacent layers, providing insight into environmental selection. The finding of organisms usually associated with anoxic sediments suggests that sulfur metabolisms associated with sediments can occur in anoxic pelagic zones and our observations expand the available known habitats for these pathways.

### New microbial agents and genes for sulfur transformation

From surface layers to the bottom, most of the genomic bins (61.4%) recovered from Lake A included genes for sulfur cycling (Figs. 3 and 4). Furthermore, bacterial abundance in Lake A was correlated with the average number of metabolic pathways for sulfur transformation per bin (*R*^2^ = 0.69, *p =* 0.04), supporting the notion that sulfur cycling represents a major process in Lake A waters and involves a large microbial diversity. Reconstruction of genomic bins highlighted that in addition to the conventional taxa associated with the classical sulfur cycle in meromictic saline lakes such as sulfate-reducing *Desulfobacteraceae*, sulfur-oxidizing *Alphaproteobacteria* and phototrophic sulfur oxidizing *Chlorobiaceae* [17], various lineages with poorly known ecological functions are also involved in sulfur transformations. Among these lineages, key genes of sulfur metabolism were identified in *Ca.* WOR1 (Saganbacteria), SAR86, *Lentisphaerae*, *Aminicentantes*, *Marinimicrobia*, *Calditrichaeota*, *Abyssubacteria*, *Omnitrophica* and *Parcubacteria*, thereby expanding the known diversity of sulfur cycling bacteria (Fig. 3 and Supplementary Table S1)*.*

A strong functional redundancy in sulfur transformation pathways was detected throughout the water column, with taxonomically diverse microorganisms having similar metabolic pathways (Figs. 3 and 4). For example, sulfide oxidation potential through SQR and the SoxABCXYZ complex was identified in *Alphaproteobacteria* (*Rhodospirillales*) and *Gammaproteobacteria* SAR86. Oxidative DsrAB genes were also identified in half of the *Rhodospirillales* bins and in the *Deltaproteobacteria* SAR324, while the modified Hdr-like complex, also involved in sulfide oxidation was discovered in another *Alphaproteobacteria* bin, congruent with experimental evidence in the *Alphaproteobacterium Hyphomicrobium denitrificans* [23]. In addition, the SoxABCXYZ complex coupled with SoeAB genes were detected in *Betaproteobacteria* bins whereas SQR, FccAB and the oxidative DsrAB genes were ascertained in the *Chlorobiaceae* bin [32] (Fig. 3). These multiple pathways for sulfide and sulfite oxidations occurred in the freshwater and chemocline layers (Fig. 4), suggesting that sulfides sustain multiple ecological niches in aquatic environments over space and time. If the occurrence of these various sulfur-oxidizing pathways and lineages at the chemocline is supported by the chemical profiles, their identification in the upper freshwater samples, coupled with the co-detection of sulfonate degradation genes (Fig. 2), suggests that organic sulfur molecules may also support sulfur-oxidizing populations in non-sulfidic waters, multiplying the availability of ecological niches and allowing functional redundancy.

In the anoxic saline waters of Lake A, the dissimilatory sulfate reduction pathway (Sat, Qmo, AprAB and DsrAB genes) occurred in the *Deltaproteobacteria* bins as expected [7], but was also found in genomic bins affiliated with *Chlorolexi*, *Planctomycetes*, *Calditrichaeota* and *Ca*. Nealsonbacteria, *Ca.* Abyssubacteria and *Ca*. Zixibacteria (Fig. 3, Supplementary Table S1). These results provide an ecological context for these new lineages of sulfate reducers that were previously proposed after mining of combined metagenomic datasets [6]. Our metagenomic survey also predicted a sulfite reduction potential (AsrAB genes) for *Omnitrophica* members (Figs. 3 and 4), confirming previous mining of publicly available metagenomes [6], as well as for few *Planctomycetes* and *Patescibacteria* populations in the sulfidic waters of the monimolimnion.

*Patescibacteria, Planctomycetes* and *Chloroflexi* phyla showed the strongest variability of genomic potential within their lineages (Figs. 3 and 4). Each of these phyla included populations predicted to gain energy from thiosulfate oxidation, sulfate and sulfite reduction as well as polysulfide/elemental sulfur reduction or oxidation. A new fusion gene probably involved in elemental sulfur/polysulfide reduction was also identified in two genomic bins affiliated with *Patescibacteria* and *Planctomycetes* phyla. Sequence comparison with public databases indicated that this gene is also present in a single-cell genome related to the *Planctomycetes*-derived phylum of the *Kiritimatiellaeota*, isolated from a deep continental microoxic subsurface aquifer [39], suggesting that this gene might be relevant in microoxic conditions. Interestingly, *Planctomycetes, Chloroflexi* and *Ca*. Nealsonbacteria (*Patescibacteria*) genomic bins also included numerous genes (> 10 per bin) coding for sulfatases. These hydrolytic enzymes potentially release sulfate from sulfated organic matter [40], providing additional electron acceptors throughout the water column. Together, these results extend the diversity of sulfur cycling microorganisms and metabolic pathways. They suggest new fundamental roles in sulfur cycling for members of the *Patescibacteria*, *Planctomycetes* and *Chloroflexi* in aquatic environments, with strong ecological niche differentiation within members of these lineages.

### Utilization of sulfur cycle intermediates

Sulfur cycle intermediates (SCIs: thiosulfate, tetrathionate, sulfite, polysulfides, elemental sulfur) have a large biogeochemical significance in anoxic and marine environments, creating shortcuts around the classic sulfur cycle [1, 8]. The potential for oxidation and reduction of these sulfur molecules was widespread in the Lake A microbial community, with taxonomically diverse lineages potentially using SCIs as electron donors or acceptors (Fig. 3). The number of genes for SCI metabolism and sulfate reduction was similar, suggesting that SCI utilisation represents a quantitatively important process in Lake A sulfur cycling (Fig. 2). Furthermore, the number of genomic bins with SCI utilization genes exceeded the number of bins with sulfate reduction and hydrogen sulfide oxidization pathways and SCIs were major hubs in the sulfur network (Fig. 5), indicating a wide diversity of microorganisms able to process SCIs.

The potential to use SCIs was shared among specialists that have the genetic potential to use only a limited range of these molecules, and generalists that could potentially metabolise a broad range of sulfur compounds including sulfate or hydrogen sulfide. The specialists included some members of the *Parcubacteria* with the potential limited to thiosulfate oxidation, *Omnitrophica* with only genes for sulfite reduction and *Bacteroidetes* populations with the metabolic potentials for thiosulfate and polysulfide oxidation. By contrast, generalists were mainly represented by members of the *Deltaproteobacteria* or *Alphaproteobacteria* lineages with a large suite of sulfur transformation genes, suggesting high variability in substrate utilization (Fig. 4).

The taxonomically diverse microorganisms observed here are likely fuelled by microbial phototrophic and chemotrophic hydrogen sulfide oxidations that generate SCIs of various oxidation states [8], as well as by abiotic oxidation of hydrogen sulfide with iron and manganese oxides present in elevated concentrations in Lake A (Fig. 1) [21]. Although SCIs have not been measured in Lake A, a sulfidic smell and a yellow-orange color of the water below 22 m was detected during sampling supporting the presence of polysulfides and aqueous elemental sulfur in the water, and the metabolic potentials detected in metagenomic dataset. Together, these results indicated a strong ecological role for SCIs by providing an energy source for a diverse and abundant microbial community in the fresh, brackish and saline waters.

### Organic sulfur molecules as SCI sources

Oxidised organic sulfur molecules, such as sulfonate and sulfonium, are produced by phytoplankton, especially dinoflagellates, as osmoprotectants, cryoprotectant, antioxidants [41] or predator deterrents in aquatic environments [42], and they are an important source of sulfur and carbon for pelagic bacteria in the ocean [14] and likely freshwaters. These compounds are even found in metabolomes of diatoms [43]. Eukaryotic microalgae including dinoflagellates such as *Polarella* and *Scrippsiella* with wide salinity tolerances have been previously reported in Lake A [28]. In addition, we detected *Chrysophyceae* and other ochrophytes (data not shown) in metagenomes from the oxic mixolimnion, and given that large eukaryotic algae would sink to greater depths, the presence of eukaryotic sulfur metabolites throughout the Lake A water column is to be expected. The genetic capacity for OSM degradation was widely distributed, occurring in 56% of the genomic bins (Figs. 3, 4 and 5). Proteobacterial lineages were detected as major sulfonate degraders in the mixolimnion and chemocline. Since these degradation processes release sulfite in the water, our results suggest that organic sulfur molecules might have an important ecological role, providing sulfur compounds of intermediate oxidation states in aerobic and microaerobic aquatic systems regardless of salinity and sulfate concentrations.

Genes for DMSP utilisation, identified in the freshwater and brackish waters of the lake were also detected in *Alphaproteobacteria* (*Rhodospirillales* and SAR11/*Pelagibacter* bins) and *Actinobacteria* (*Acidimicrobiia*), as reported in surface oceans [14]. In addition, numerous genes for respiration of dimethylsulfoxide (DMSO) were identified in the anoxic monimolimnion and in *Desulfobacteraceae*, *Chloroflexi* and *Bacteroidetes* bins, suggesting that algal metabolites could sink within senescent phytoplankton cells from the upper water column and then be used as an alternative energy source by anaerobic microbial populations in the lower water column.

## Conclusions

Isolated and permanently stratified, High-Arctic meromictic saline Lake A offered an ideal opportunity to uncover and dissect the pathways of microbial sulfur metabolism across oxygen, sulfate, sulfide and salinity gradients. Although the chemocline harboured the taxonomically and functionally most diverse and abundant microbial community, demarcating this layer as a hotspot for microbial activity and sulfur transformations as in other meromictic lakes [17, 19], genes for sulfur transformations were identified throughout the water column, and provided new insights into sulfur cycling by microorganisms over an unusually wide range of environmental conditions. The pathways and taxa involved in sulfide oxidation and sulfate reduction were complex and diverse. However, the metagenomic dataset revealed that sulfur transformations were not limited to these classic processes and that multiple sulfur cycling pathways may be simultaneously operating throughout the water column, with taxonomically diverse populations using sulfur cycle intermediates as electron donors or acceptors. Genes for organic sulfur molecule degradation and respiration were also abundant and widely distributed in the microbial community, suggesting that phytoplankton metabolites might also be a major source of energy for freshwater and marine bacteria. Our data extend the diversity of sulfur cycle lineages and metabolic pathways in aquatic ecosystems and emphasise the ecological importance of sulfur cycle intermediates as key hubs for electron flow and energy production over a wide range of environmental conditions.

## Methods

### Sample collection and nucleic acid extraction

In summer 2017 (18 July 2017), three independent 24-cm-diameter holes were drilled through the ice (0.6 m thickness) near the middle of Lake A (Ellesmere Island, 82° 59.667′ N, 75° 26.602′ W; Fig. 1a). Oxygen concentration, salinity and temperature profiles throughout the water column were measured using a YSI-EXO2 profiler (Xylem, Inc., Yellow Springs, USA) to 65 m, which was the length of the instrument cable, to determine sampling depth. Eight sampling depths (2, 6, 14, 22, 34, 40, 55 and 65 m) were selected to cover all identified water layers (Fig. 1). A Limnos water sampler (KC, Denmark), which was previously rinsed with 10% HCl, then sterile MilliQ water and maintained closed until sampling, was lowered into the ice holes down to the sampling depth. Water was visibly orange in the 22 m, 34 m, 40 m, 55 m and 65 m samples. For each selected depth, 1 L of each triplicate water sample was directly filtered through separate 0.22-μm pore size Sterivex filters and then stored below – 50 °C until nucleic acid extraction.

Nucleic acids (DNA and RNA) of two of the replicates samples per depth were extracted from the same Sterivex filters using Qiagen Allprep DNA/RNA Mini Kit. The Sterivex cartridges were opened and the membrane filters were cut into small pieces before the lysis steps, as previously described [44]. All steps of the nucleic acid extractions, from the opening of the filters to the nucleic acid resuspension in autoclaved, filtered and UV-treated water, were carried out in a sterile laminar flow cabinet. Negative control (no template) of nucleic acids extraction was simultaneously carried out. The DNA extracts were stored at – 20 °C until library preparation. For RNA extracts, two additional DNase steps (DNase I, Ambion, Foster City, CA, USA) were carried out to remove any trace of carried over DNA. The absence of DNA contamination was confirmed by amplification of 16S rRNA genes with bacterial primers using the RNA extracts (undiluted and diluted 10 times) as template, with no product detected after 35 PCR cycles. The RNA was then immediately converted to cDNA using a High-Capacity cDNA Reverse Transcription kit (Applied Biosystems, Foster City, CA, USA) and stored as cDNA at − 20 °C until library preparation.

### Quantitative PCR

The abundance of bacterial and archaeal 16S rRNA genes was estimated on the two replicate samples per depth using quantitative PCR (qPCR) with primers Bact1369f/Bact1492r [45] and Arc787f/Arc1059r [46], respectively. Quantification was performed in triplicate with a range of template concentrations (0.1, 0.5, 1 ng of DNA) to compensate for any PCR inhibition. Genomic DNA extracted from *Methylomonas methanica* (DSM25384) and *Methanosarcina acetivorans* (DSM2834) were serially diluted to construct standard curves (concentration ranged from 10^2^ to 10^6^ 16S rRNA genes per reaction). The *R*^2^ of standard curves obtained by qPCR were above 0.99, PCR efficiencies were above 88.7%, and melting curves showed no trace of non-specific amplifications. Threshold cycle (Ct) of the samples ranged from 15 to 25 cycles whereas Ct of negative controls (water) were all after 37 cycles. The qPCR results were expressed in terms of 16S rRNA gene numbers per millilitre of water sample (Fig. 1d).

### Illumina MiSeq Amplicon library preparation, sequencing and analysis

Microbial community composition of the samples was determined by high-throughput sequencing of bacterial and archaeal 16S rRNA (cDNA) and 16S rRNA genes (DNA) using primers targeting the bacterial V4–V5 region (S-D-Bact-0516-a-S-18/S-D-Bact-0907-a-A-20; 460 bp product) [47] and the archaeal V1–V3 region (A27F/Arc518R; 500 bp), respectively [48]. All PCR reactions were carried out following [49]. Samples and negative controls of nucleic acids extraction, transcription and PCRs were sequenced using an Illumina MiSeq v3 kit at the IBIS/Laval University, Plate-forme d’Analyses Génomiques (Québec, QC). Reads were assembled into single paired-end sequences, curated and clustered into OTUs (97% sequence similarity) as detailed in a GitHub repository (https://github.com/CruaudPe/MiSeq_Multigenique). OTUs detected in negative controls were removed from the analysis as described [27]. Taxonomic affiliations of the reads were determined with Mothur [50] using BLAST against Silva database release 132 as reference [51].

### Metagenomic library preparation, sequencing and analysis

One metagenome per sample depth (8 metagenomes) was constructed using a Nextera XT Library Kit (Illumina, San Diego, CA, USA). The 8 metagenomes were pooled equimolarly then sequenced in two Illumina MiSeq (2 × 300 bp) runs and one Illumina NexSeq run (2 × 150 bp) at the Institut de Biologie Integrative et des Systèmes (IBIS) sequencing platform (Université Laval, Canada) and at the CGEB–Integrated Microbiome Resource (Dalhousie University, Canada), respectively. Datasets were quality filtered using the Trimmomatic tool [52], with default settings. Paired-end joining was done using FLASH2 [53]. The 16S rRNA reads longer than 110 bp were isolated from metagenomic reads using REAGO 1.1 [54], and taxonomic assignments were performed as for the 16S rRNA gene amplicons.

Each metagenome was assembled separately from paired-end reads passing quality filtering using SPAdes [55]. Assembled contigs and mapping files (BAM files generated using BBmap) were uploaded to the Department of Energy Joint Genome Institute (DOE-JGI) IMG/MER analysis pipeline [56] for gene calling and functional annotation. To account for differences in sequencing depth between samples, metagenomes were normalised to the size of the smallest dataset.

### Binning and functional characterization

For metagenome assembled genome reconstruction, all quality filtered sequences were pooled and co-assembled using MEGAHIT [57]. Read coverage of the contigs was carried out using bwa-mem (http://bio-bwa.sourceforge.net), followed by contig binning using MetaBAT-2 [58] with contigs longer than 2000 bp. The completeness and contamination level of the combined genomic bins were then evaluated using CheckM [59]. Only bins with a contamination level under 5% and completeness above 50% were analysed. Genetic composition of genomic bins was then explored using KEGG [25] and MetaCyc [60] pathway mappers with genes identified by IMG/MER in the co-assembly. The results were checked manually and the presence of specific pathways not characterised in the KEGG database was determined using BLASTP with parameters detailed in the notes for Supplementary Table 1. In addition, presence and affiliation of genes coding molybdenum enzymes (DMSO reductase family) was investigated using GraftM and its DMSO superfamily package [61]. Taxonomic affiliation of the bins was based on 16S rRNA gene when present in the bin as well as on taxonomic affiliation and phylogenetic analysis of ribosomal protein genes. Protein sequence alignments were performed using Clustal Omega [62] and phylogenetic tree of concatenated sequences of ribosomal protein alignment and otr protein sequence alignment were conducted using FastTree 2 [63] with the JTT+CAT model and visualised using iTOL [64].

### Statistical analysis

Statistical analyses of the data set (Student’s *t* test, non-parametric multivariate analysis of variance (NPMANOVA), Bray-Curtis-based dissimilarity index calculations and correlation-based clustering, non-metric multidimensional scale (NMDS)) were carried out according to recommendations of the Guide to Statistical Analysis in Microbial Ecology [65], using PAST software [66]. Sulfur cycling network was represented using the software environment R implemented with the igraph package [67].

## Supplementary Information

**Additional file 1: Supplementary Figure S1.** Heatmap representing the bacterial community composition in each sample determined using 16S rRNA gene (D), 16S rRNA (R) and 16S rRNA reads recovered from metagenomic data (M). Results of the two duplicate samples are shown for 16S rRNA gene and 16s rRNA. Only lineages with relative abundance >1% in at least one sample are shown. The right part of the graph represents the bacterial 16S rRNA gene quantification in the duplicate samples (blue bars), as well as the relative proportion between bacteria and archaea determined by 16S rRNA gene qPCR quantification (D; red bars) and in the metagenomic dataset (M; purple bar).

**Additional file 2: Supplementary Figure S2.** Relative proportion of bacterial lineages obtained with the different molecular approaches a) Relative proportion of bacterial lineages observed in the rRNA gene amplicon dataset and rRNA amplicon dataset. b) Relative proportion of bacterial lineages observed in the rRNA gene amplicon dataset and in the metagenomic dataset. Each point represents a bacterial lineage as defined in Supplementary Figure S1. Points are color coded according to their sample of origin.

**Additional file 3: Supplementary Figure S3.** Phylogenetic tree of the octaheme c-type cytochrome tetrathionate reductase genes (Otr). The tree is rooted with octaheme c-type cytochrome nitrite reductase.

**Additional file 4: Supplementary Figure S4.** Phylogenetic tree of the genomic bins. The tree is based on concatenated alignment of ribosomal protein genes. Only bins with more than 50% of the ribosomal protein genes are included in the tree. The branches are color coded according to the taxonomy in Fig. 1.

**Additional file 5: Supplementary Table S1.** Description of the genomic bins with sulfur-cycling genes identified in the Lake A microbiome presented in Figure 3.

## Data Availability

Assembled metagenome data are available in IMG/MR (https://img.jgi.doe.gov/mer/) under the following accession numbers: 3300033443, 3300033444, 3300033445, 3300033439, 3300033411, 3300033473, 3300033474 and 3300033495. Co-assembly is also available on IMG/MR under accession number 3300033064. Raw amplicon sequences and bin files were deposited in the NCBI public database under Bioproject PRJNA616293 (https://www.ncbi.nlm.nih.gov/bioproject/PRJNA616293). In-house scripts used in this study are available on GitHub/CruaudPe. Environmental metadata were previously published [21] and additional data are available in the Nordicana D database (http://www.cen.ulaval.ca/nordicanad).

## Abbreviations

S: Sulfur
OSM: Organo-sulfur molecules
SCI: Sulfur cycle intermediate
DMS: Dimethylsulfide
DMSP: Dimethylsulfoniopropionate
DMSO: Dimethylsulfoxide
OTU: Operational taxonomic unit
qPCR: Quantitative polymerase chain reaction
NMDS: Non-metric multidimensional scaling
NPMANOVA: Non-parametric multivariate analysis of variance
MSBL: Mediterranean Sea Brine Lake
Ca.: Candidatus
BLAST: Basic Local Alignment Search Tool
PAR: Photosynthetically available radiation
