# Supplementary figures and images for: Genomic evidence for sulfur intermediates as new biogeochemical hubs in a model aquatic microbial ecosystem
Source: Microbiome. 2021 Feb 16;9:46. doi: 10.1186/s40168-021-00999-x (PMC7887784; doi:10.1186/s40168-021-00999-x)

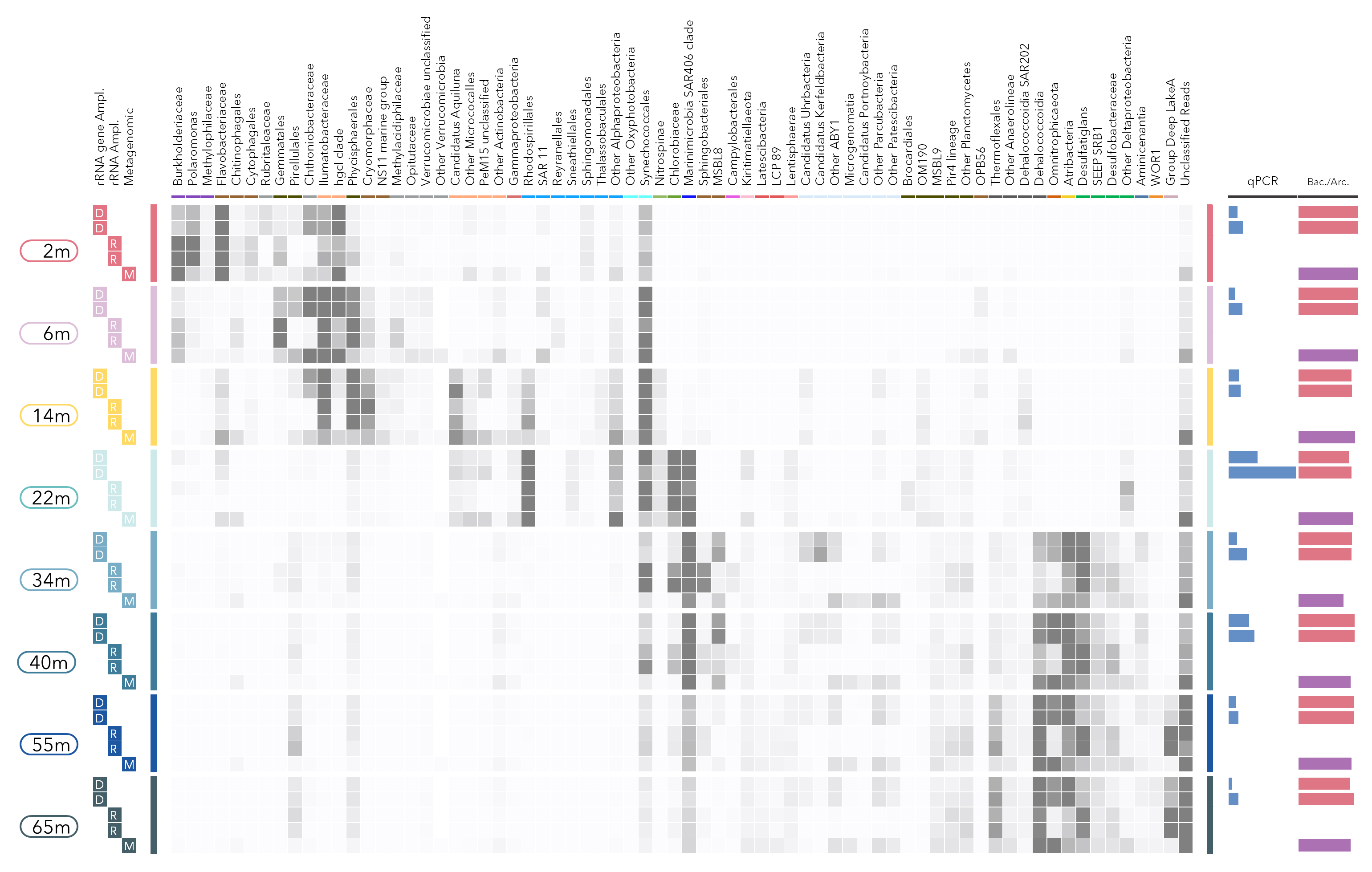

Supplement: Supplementary file 2 — Additional file 1: Supplementary Figure S1. Heatmap representing the bacterial community composition in each sample determined using 16S rRNA gene (D), 16S rRNA (R) and 16S rRNA reads recovered from metagenomic data (M). Results of the two duplicate samples are shown for 16S rRNA gene and 16s rRNA. Only lineages with relative abundance >1% in at least one sample are shown. The right part of the graph represents the bacterial 16S rRNA gene quantification in the duplicate samples (blue bars), as well as the relative proportion between bacteria and archaea determined by 16S rRNA gene qPCR quantification (D; red bars) and in the metagenomic dataset (M; purple bar). [file 40168_2021_999_MOESM2_ESM.tif]

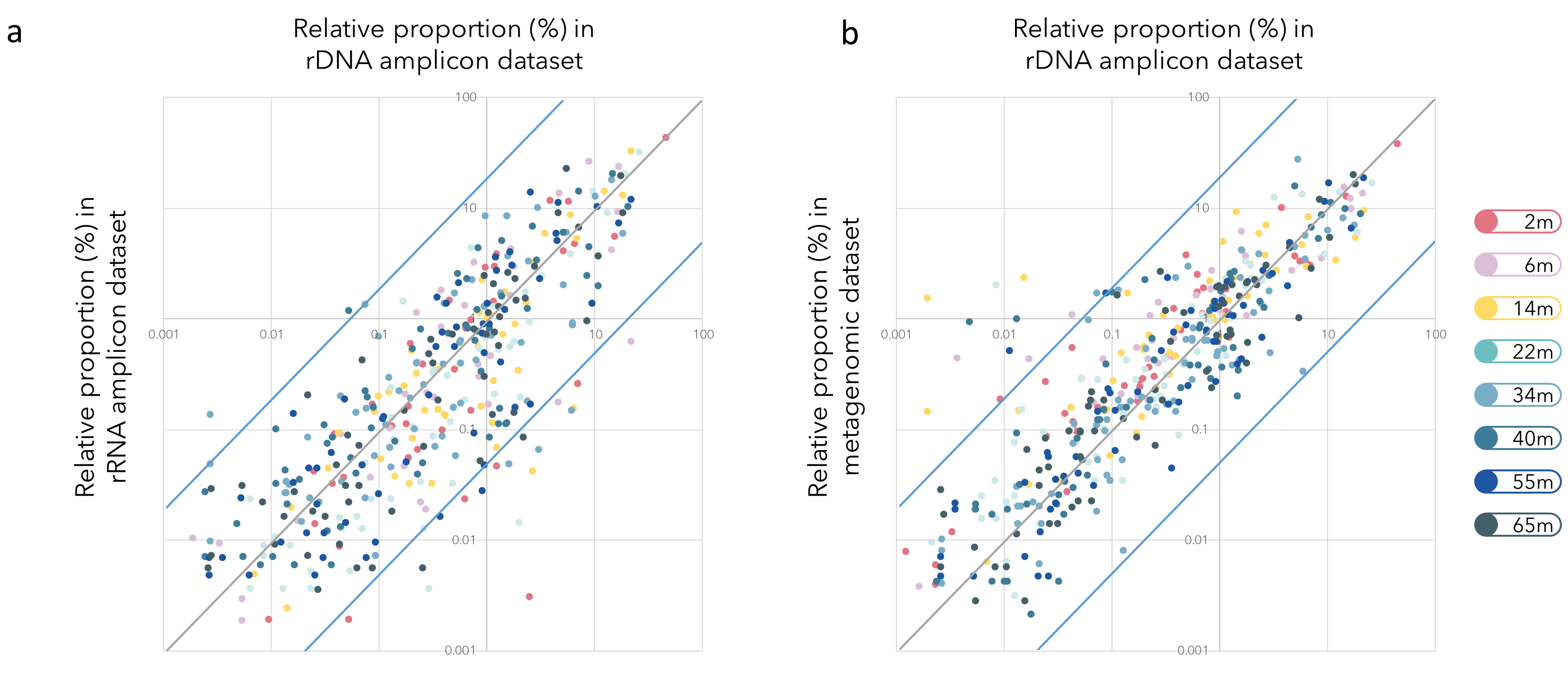

Supplement: Supplementary file 3 — Additional file 2: Supplementary Figure S2. Relative proportion of bacterial lineages obtained with the different molecular approaches a) Relative proportion of bacterial lineages observed in the rRNA gene amplicon dataset and rRNA amplicon dataset. b) Relative proportion of bacterial lineages observed in the rRNA gene amplicon dataset and in the metagenomic dataset. Each point represents a bacterial lineage as defined in Supplementary Figure S1. Points are color coded according to their sample of origin. [file 40168_2021_999_MOESM3_ESM.tif]

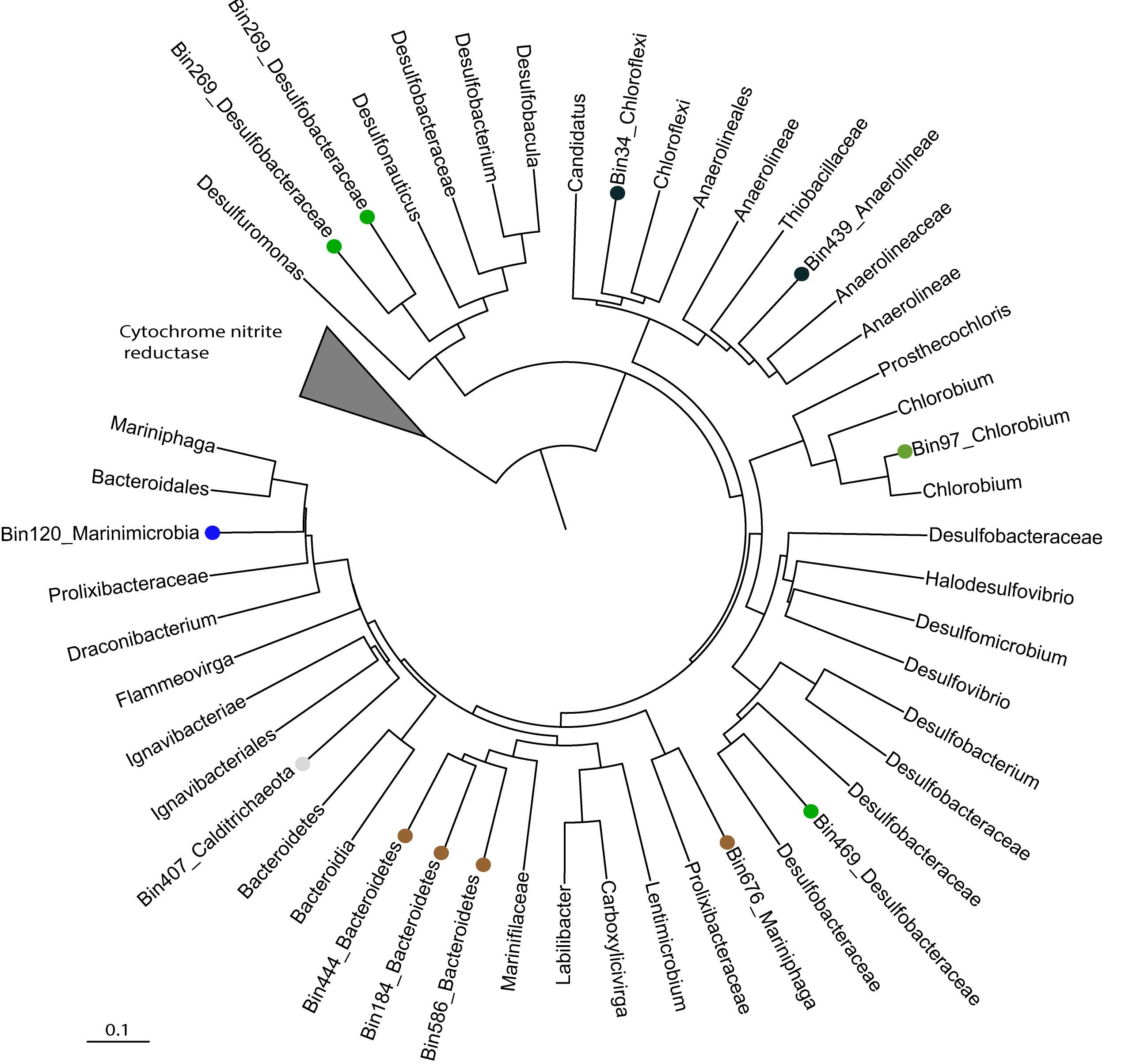

Supplement: Supplementary file 4 — Additional file 3: Supplementary Figure S3. Phylogenetic tree of the octaheme c-type cytochrome tetrathionate reductase genes (Otr). The tree is rooted with octaheme c-type cytochrome nitrite reductase. [file 40168_2021_999_MOESM4_ESM.tif]

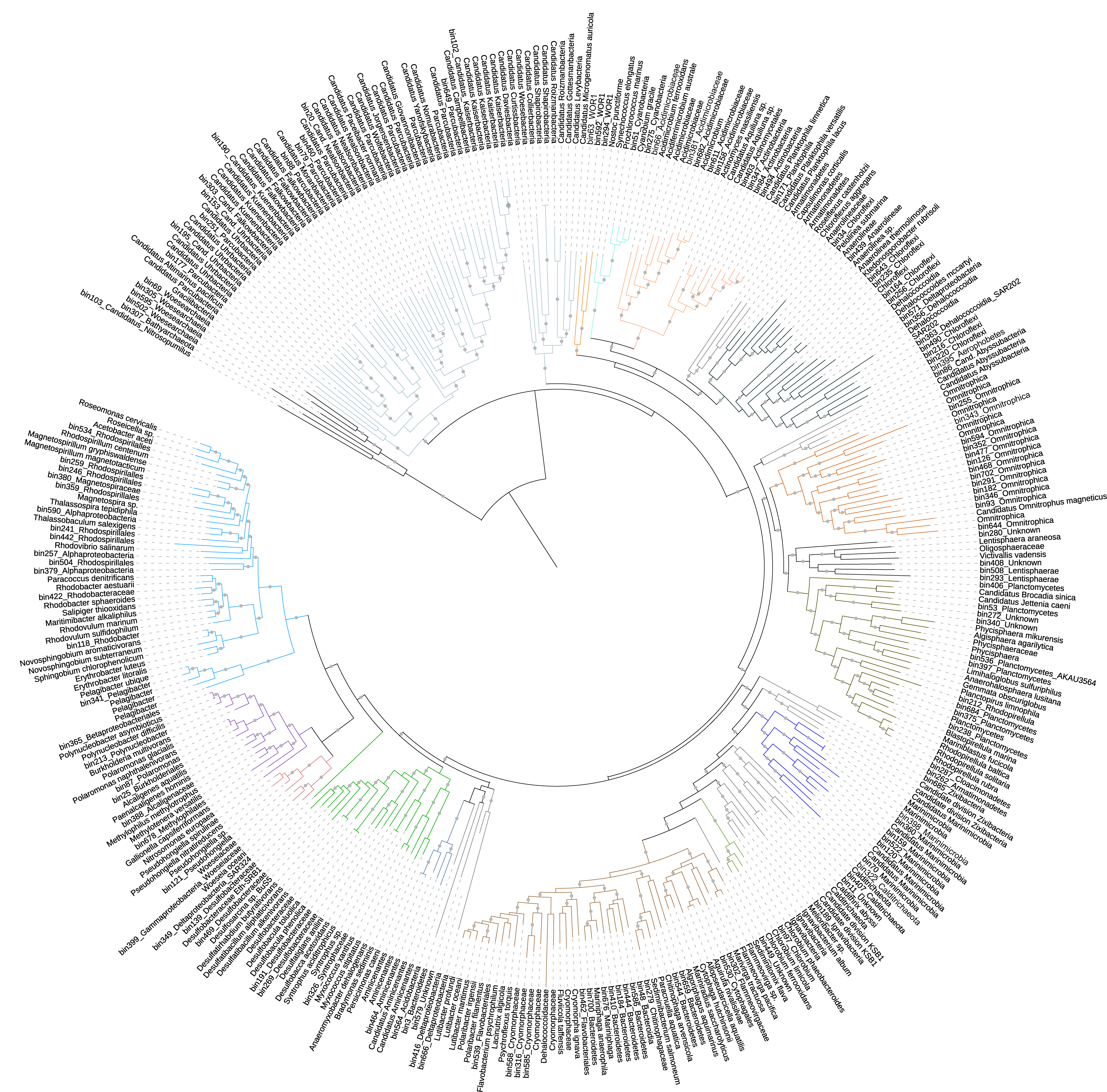

Supplement: Supplementary file 5 — Additional file 4: Supplementary Figure S4. Phylogenetic tree of the genomic bins. The tree is based on concatenated alignment of ribosomal protein genes. Only bins with more than 50% of the ribosomal protein genes are included in the tree. The branches are color coded according to the taxonomy in Fig. 1. [file 40168_2021_999_MOESM5_ESM.tif]
